# Editorial: The art of teaching physiology: its place in the integrated curriculum

**DOI:** 10.3389/fphys.2023.1334822

**Published:** 2023-12-08

**Authors:** Amberly Reynolds, Irina Nizamutdinova

**Affiliations:** ^1^ Rocky Vista University, Ivins, UT, United States; ^2^ University of the Incarnate Word School of Osteopathic Medicine, San Antonio, TX, United States

**Keywords:** education, physiology, intergration, curricula, medical educaion

Medical education is at a crossroads in which faculty, leadership, and students are charging forward with a mission to integrate the pre-clinical curriculum better. In our current state, courses are often offered in parallel but not integrated; for example, anatomists work predominately on educating students on the structure of the human body and may touch on basic physiology, but complete physiological education comes from an independent physiology course. The other familiar layout of the curriculum is to have a systems-based approach in which the content is integrated, but integrated means an anatomy lecture on Monday and a physiology lecture on Tuesday. Overall, the full integration is missing. Thus, we were interested in working with Frontiers in Physiology to produce this special edition in which we asked contributors to research The Art of Teaching Physiology, explicitly focusing on integrating the curriculum.

We are excited for you to read the perspective article from Slater et al., which addresses a new osteopathic medical school’s work on integrating physiology into the undergraduate medical education curriculum. They integrate several disciplines in a session. They describe the development and implementation process, which may prove invaluable to new and older medical programs when considering integrative curricula. Dennis and Creamer provide a brief research report on linking physiology with the anatomical areas of histology and embryology via foundational science. Allison et al. focus on module development within the gross anatomy lab. Here, they were able to use modules to bring anatomy and physiology together for students under the banner of structure and function through the support of an anatomy laboratory environment. Looking to specifics on a smaller scale, Bassey et al. provide an article that focuses on the cardiovascular block at their institution. They used spiral integration to place physiologic concepts into the 5-week cardiovascular block during the Homeostasis course. LeClair et al. provide a perspective on their goal of creating a pre-clerkship curriculum devoid of disciplinary boundaries. They focus on the concept that physicians use illness scripts (integrated cognitive schema) with integrated content knowledge. Additionally, we highlight the research by Xu et al. as they worked to identify author collaborations and impact with analysis of research hotspots and frontiers in teaching reforms within physiology education.

We are proud of our colleagues and their articles, which provide perspective, methodologies, and further guidance on working to integrate physiology within curricula, specifically within medical education. As medical education continues to grow and change to meet the standards, we also look to the students and faculty for guidance on best practices and curricular reform. Through research like that presented by her and with Frontiers in Physiology, we are primed to learn lessons that will benefit medical education for years.

